# Prevention of Catheter-Related Bacteremia with a Daily Ethanol Lock in Patients with Tunnelled Catheters: A Randomized, Placebo-Controlled Trial

**DOI:** 10.1371/journal.pone.0010840

**Published:** 2010-05-26

**Authors:** Lennert Slobbe, Jeanette K. Doorduijn, Pieternella J. Lugtenburg, Abdelilah el Barzouhi, Eric Boersma, Willem B. van Leeuwen, Bart J. A. Rijnders

**Affiliations:** 1 Department of Internal Medicine, Division of Infectious Diseases and Department of Medical Microbiology and Infectious Diseases, Erasmus Medical Center, Rotterdam, The Netherlands; 2 Department of Haematology, Erasmus Medical Center, Rotterdam, The Netherlands; 3 Department of Cardiology, Erasmus Medical Center, Rotterdam, The Netherlands; Oregon Health and Science University, United States of America

## Abstract

**Background:**

Catheter-related bloodstream infection (CRBSI) results in significant attributable morbidity and mortality. In this randomized, double-blind, placebo-controlled trial, we studied the efficacy and safety of a daily ethanol lock for the prevention of CRBSI in patients with a tunnelled central venous catheter (CVC).

**Methodology:**

From 2005 through 2008, each lumen of the CVC of adult hematology patients was locked for 15 minutes per day with either 70%-ethanol or placebo, where after the lock solution was flushed through. As a primary endpoint, the incidence rates of endoluminal CRBSI were compared.

**Principal Findings:**

The intent-to-treat analysis was based on 376 patients, accounting for 448 CVCs and 27,745 catheter days. For ethanol locks, the incidence of endoluminal CRBSI per 1000 CVC-days was 0.70 (95%-CI, 0.4–1.3), compared to 1.19 (95% confidence interval, 0.7–1.9) for placebo (incidence rate-ratio, 0.59; 95% confidence interval, 0.27–1.30; P = .19). For endoluminal CRBSI according to the strictest definition (positive hub culture and identical bacterial strain in blood), a 3.6-fold, non-significant, reduction was observed for patients receiving ethanol (2 of 226 versus 7 of 222; P = .103). No life-threatening adverse events were observed. More patients receiving ethanol discontinued lock-therapy (11 of 226 versus 1 of 222; P = .006) or continued with decreased lock-frequency (10 of 226 versus 0 of 222; P = .002), due to non-severe adverse events.

**Conclusions:**

In this study, the reduction in the incidence of endoluminal CRBSI using preventive ethanol locks was non-significant, although the low incidence of endoluminal CRBSI precludes definite conclusions. Therefore, the lack of statistical significance may partially reflect a lack of power. Significantly more patients treated with ethanol locks discontinued their prophylactic treatment due to adverse effects, which were non-severe but reasonably ethanol related. Additional studies should be performed in populations with higher incidence of (endoluminal) CRBSI. Alternative sources of bacteremia, like exoluminal CRBSI or microbial translocation during chemotherapy-induced mucositis may have been more important in our patients.

**Trial Registration:**

ClinicalTrials.gov NCT00122642

## Introduction

The indwelling central venous catheter (CVC) has become an essential feature of modern patient management. However, its use puts patients at risk for various complications, especially catheter-related bloodstream infection (CRBSI). CRBSI accounts for a major cause of healthcare-related bacteremia and leads to prolonged hospital stay and significant attributable costs.[1]–[4] Reported attributable mortality varies from 2% up to 25% in critically ill patients.[2], [5] In a meta-analysis, the odds-ratio for mortality in patients with CRBSI was 1.65 compared to control patients who were matched for severity of illness.[5]

In contrast to short-term CVCs, CRBSI in patients with tunnelled or implanted devices is thought to be mainly caused by endoluminal colonization due to contamination of the catheter hub.[6], [7] Evidence-based recommendations on CRBSI prevention have been published.[8], [9] To some extent, endoluminal CRBSI can be prevented if an *antibiotic* solution is instilled in the catheter.[10]–[12] However, the preventive use of antibiotics should be avoided if alternative options exist.[13], [14] Although there is evidence to support the concept, methodologically appropriate clinical studies on the use of preventive *antiseptic* solutions are scarce. For this purpose, ethanol is increasingly considered as a promising candidate. For CRBSI-*treatment*, an ethanol-lock has been demonstrated to be efficacious in several observational studies.[15]–[17] More recently, an ethanol-lock has also been studied for CRBSI-*prevention*.[18]–[21] A major advantage of ethanol would be the broad antimicrobial spectrum without compromising future antibiotic treatment. Furthermore, it is cheap and universally available.

In the current randomized, clinical trial, we study the efficacy and safety of a daily 70%-ethanol lock on the prevention of endoluminal CRBSI in hematology patients with long-term tunnelled catheters.

## Materials and Methods

The protocol for this trial and supporting CONSORT checklist are available as supporting information; see Protocol S1 and Checklist S1.

### Ethics statement

The institutional review board approved the protocol; written informed consent was obtained from all patients.

### Study design and ethanol lock procedure

The study was performed at the Erasmus Medical Center, a tertiary referral hospital with 2 locations in Rotterdam, The Netherlands. Eligible study-participants were all consecutive adult (age, >17 years) hematology patients with a tunnelled silicone CVC, inserted in the preceding 72 hours before study-entry. All catheters used in this study, were silicone devices (Hickman®), that were placed at the radiology ward after carefully scrubbing the insertion site with chorhexidine-containing antiseptics, applying maximum sterile barrier precautions, including the use of a long-sleeved sterile gown, cap, mask and gloves, together with the use of sterile sheet drapes.

Excluded were patients with an alcohol-intolerance or concomitant treatment with metronidazole. Patients were enrolled from July 2005 through August 2008. The study was a randomized, double-blind, placebo-controlled trial (ClinicalTrials.gov Identifier: NCT00122642). Randomization was performed using a computer-generated list of randomly assigned permuted blocks. Randomization was catheter-based, which implies that patients could be randomized more than once if insertion of a new CVC was needed. Concealment of allocation and provision of blinding was guaranteed by uninvolved employees of the Department of Pharmacy, who delivered patient-labelled ampoules containing either 70%-ethanol or placebo (0.9% NaCl).

During hospitalization, every lumen of the CVC (3 ml) was locked for 15 minutes per day, following which the solution was flushed through with 10 ml 0.9% NaCl. During outpatient settings, the lock was instilled by the nursing staff once weekly before renewal of the regular heparin solution.

An investigator-blinded safety analysis was performed after inclusion of 80 patients, as some patients experienced adverse effects immediately after flushing the lock solution through. This provisional analysis led to an amendment that allowed these patients to continue with a modified lock regimen, in which only 1 lumen was locked per day.

### Data collection and definitions

Baseline characteristics included age, sex, presence of neutropenia (neutrophil count, <500 cells/µl) at study-entry, underlying malignancy, site of catheter insertion, and number of catheter lumens. During follow-up, we recorded catheter dwell time, stay at the intensive care unit, treatment with total parenteral nutrition (TPN), and use of glycopeptides, which is the treatment for presumed/proven beta-lactamase resistant gram-positive microorganisms in our hospital. Safety data were registered for all patients, including all-cause mortality during the study episode until 30 days after catheter removal, and the discontinuation of study medication. Subjective parameters were recorded for a random sample of 25% of patients by means of a questionnaire.

In case of suspected CRBSI, definitions from current guidelines were followed.[8] In case of documented bacteremia or when a glycopeptide was started empirically, a culture of the inside of all catheter hubs was performed. The catheter insertion site was cultured in case of local inflammation or unexplained bacteremia. As our intervention would reasonably prevent only endoluminal CRBSI, effort was made to distinguish this modality. Strictly *endoluminal* CRBSI was defined as a positive central or peripheral blood culture with the same genotypic (for coagulase-negative staphylococci (CNS)) or phenotypic (for other microorganisms) strain cultured from the hub, for which directed antimicrobial therapy was started. For CNS or other skin-colonizers, ≥2 blood cultures had to be positive when no peripheral cultures were available.

In case of bacteremia, we calculated the differential time-to-positivity (DTTP), which denotes the difference in time-to-positivity of a peripheral blood culture minus the time-to-positivity of a central blood culture. DTTP of ≥2 hours accurately predicts the catheter to be the source of the episode of bacteremia, which applies especially to patients with long-term catheters.[22], [23] Therefore, CRBSI with DTTP of ≥2 hours in the absence of insertion site or tunnel infection was considered as a separate entity. When DTTP was not available, CRBSI was diagnosed in case of a positive peripheral or central blood culture with an identical microorganism detected on the catheter tip in the absence of any other infectious source. The latter two entities were defined as presumed endoluminal CRBSI, because strictly, these episodes cannot be diagnosed as endoluminal CRBSI with absolute certainty, although an endoluminal origin is more likely in the absence of signs of exoluminal infection. *Exoluminal* CRBSI was defined as bacteremia with negative hub cultures, but with the same strain cultured from blood and a clinically infected insertion site, combined with either DTTP of ≥2 hours or an identical microorganism detected on the tip.

### Microbiological procedures

Regardless of suspicion of infection, catheter tips were processed by the semi-quantitative roll plate method.[24] After incubation for 72 hours, microorganisms were identified and quantified by standard microbiological methods. Catheter tip colonization was defined as a positive semi-quantitative tip culture of ≥15 colony forming units (cfu)/ml. Blood cultures were processed according to routine procedures, using the Bactec system (BD; USA).

For genetic typing of isolated CNS strains, we used arbitrarily-primed PCR, as described in detail elsewhere.[25], [26] Strains were considered identical if all 3 primers showed corresponding DNA-fingerprints. When the strain was not available for genotyping, strains were considered phenotypically identical if antibiotic susceptibility patterns showed at maximum one disconcordant result.

### Outcome

End points were reviewed by 2 blinded investigators (L.S. and B.J.R.). Patients with strictly endoluminal CRBSI and patients with presumed endoluminal CRBSI were considered as the primary outcome measure. The predefined secondary goals were to compare overall CRBSI (including exoluminal infection), overall bacteremia, incidence of positive hub and catheter tip cultures, all-cause mortality, and treatment with systemic antibiotics (glycopeptides versus other compounds) for both groups. Safety data were also assessed as a secondary outcome.

### Statistics

Statistical analyses were performed using SPSS, version 15.0 (Chicago; USA). Tests were two-sided and a P value <.05 was considered statistically significant. Analyses were based on catheter episodes and performed on a modified intent-to-treat (ITT) population, consisting of all enrolled patients who received at least 1 dose of study-solution. Follow-up was censored at the moment a primary endpoint was diagnosed, at catheter removal, or at death of patients.

Based on a comparable population, CRBSI was assumed to occur in ≥20%.[27] We assumed that the majority of CRBSI would be endoluminal CRBSI. Therefore, a sample size of 219 catheter episodes per group was calculated to detect a hypothesized 50%-reduction of endoluminal CRBSI with 80% power (α = .05). According to recommendations of the Centers for Disease Control and Prevention, we also determined CRBSI rates per 1000 CVC-days.[28] Kaplan-Meyer curves, to describe the rate of CRBSI for both groups as a function of time, were constructed and compared with log-rank tests.

The separate contribution of TPN, stay at the intensive care unit, underlying disease, neutropenia at time of catheter insertion, catheter insertion site, and number of catheter lumens were assessed in a Cox regression model. For secondary endpoints, differences between groups were analyzed with chi-square tests or Fisher's exact tests in case of dichotomous variables; differences in means were compared with student's t-tests, as appropriate.

## Results

**Figure 1:**
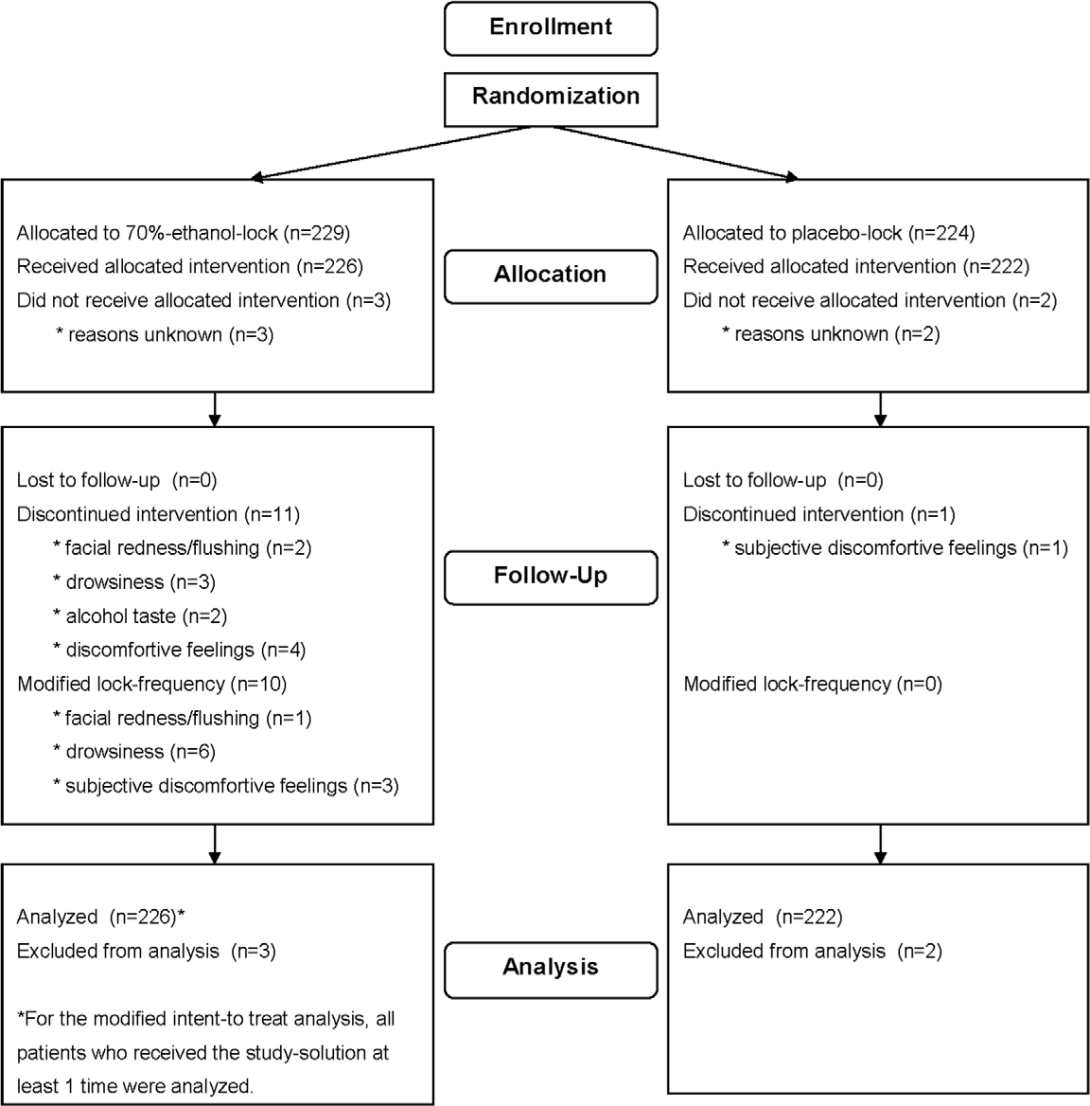
Flow-diagram for numerical illustration of the different stages of the study.

A total of 379 patients were enrolled, accounting for 453 catheter episodes. No study solution was administered in 5 catheter episodes, so the modified ITT-analysis was based on data obtained from 448 episodes (376 patients). Ethanol locks were administered in 226 catheter episodes. Characteristics of the 2 groups are summarized in Table 1; a flow-diagram of the study protocol is provided in Figure 1.

**Table 1:** Patient characteristics.

			Ethanol	Placebo
			(n = 226)	(n = 222)
**Baseline**				
	Age, mean years (range)		51.7 (18–75)	49.8 (18–74)
	Male sex		130 (57.5)	125 (56.3)
	Neutropenia^a^ at insertion		44 (19.5)	47 (21.2)
	Underlying malignancy			
		AML-MDS or ALL	140 (61.9)	119 (53.6)
		Other	86 (38.1)	103 (46.4)
	Type of central venous catheter			
		Double-lumen	83 (36.7)	99 (44.6)
		Triple-lumen	139 (61.5)	122 (55.0)
		Missing data	4 (1.8)	1 (0.4)
	Insertion place			
		Internal jugular vene	214 (94.7)	218 (98.2)
		Subclavian vene	5 (2.2)	0 (0.0)
		Femoral vene	1 (0.4)	1 (0.4)
		Missing data	6 (2.7)	1 (0.4)
**Follow-up**				
	Catheter dwell time, mean days (range)		63.1 (2–486)	60.7 (4–308)
	Total parenteral nutrition		117 (51.8)	91 (41)
	Stay at intensive care unit		18 (8.0)	13 (5.9)

Data represent numbers (%) of patients unless indicated otherwise. AML-MDS, acute myeloid leukemia-myelodysplastic syndrome; ALL, acute lymphoblastic leukemia.

a Neutrophil count, <500 cells/µl.

### Prophylactic effect of 70%-ethanol lock on CRBSI

**Figure 2:**
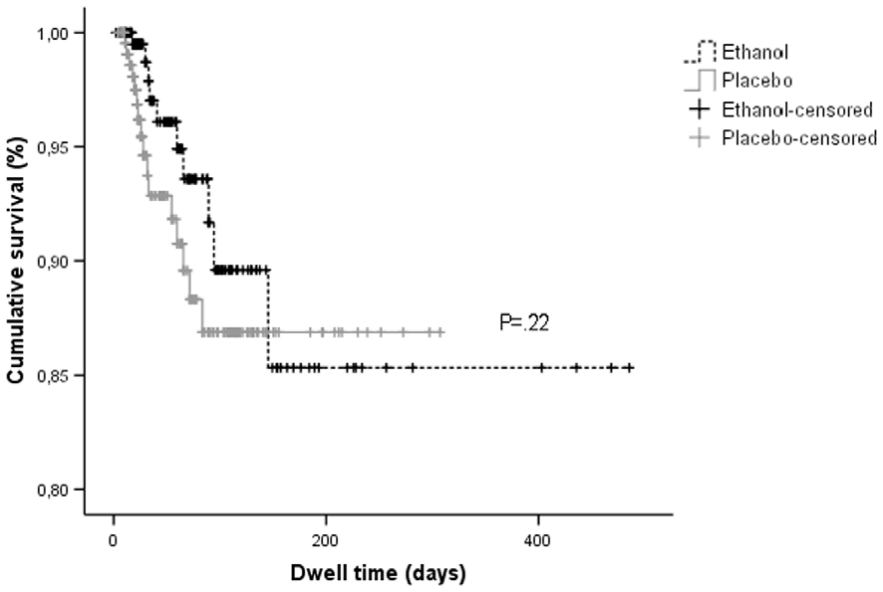
Kaplan-Meyer survival curves for comparison of the rate of catheter-related bloodstream infections. Data are presented as a function of catheter dwell time for patients treated with an ethanol lock (n = 226) or placebo.

The differences between the rates of endoluminal CRBSI in both groups were not statistically significant (Table 2). For ethanol locks, a total of 14,262 catheter days and 10 episodes of endoluminal CRBSI were recorded, accounting for a rate of 0.70 CRBSIs per 1000 CVC-days (95% confidence interval, 0.4–1.3). For placebo, 16 endoluminal CRBSIs during 13,483 catheter days were observed, with a rate of 1.19 CRBSIs per 1000 CVC-days (95% confidence interval, 0.7–1.9). The calculated incidence rate ratio was 0.59 (95% confidence interval, 0.27–1.30), which implies a non-significant reduction of 41% for patients treated with ethanol locks (P = .19). In Figure 2, Kaplan-Meyer curves are presented to describe the rates of CRBSI as a function of catheter dwell time. No significant difference was observed when these curves were compared with log-rank tests (P = .22). In patients who classified for endoluminal CRBSI according to the strictest definition (positive hub culture with identical bacterial strain in blood), a 3.6-fold reduction was observed for patients allocated to ethanol locks (2 of 226 versus 7 of 222; P = .103).

**Table 2:** Overview of endpoints and other parameters.

	Ethanol	Placebo	P
Parameter	(n = 226)	(n = 222)	
Strictly endoluminal CRBSI	2	7	.10
Presumed endoluminal CRBSI	8	9	.81
Combined primary endpoint	10	16	.23
Primary bacteremia	91	91	.95
Positive culture of catheter hub^a^	8	11	.67
Positive culture of catheter tip^b^	49	57	.52
Exoluminal CRBSI	11	8	.64

Data represent numbers of events. CRBSI, catheter-related bloodstream infection.

a Positive catheter hub cultures, performed during episodes of bacteremia (n = 73 for ethanol; n = 74 for placebo).

b Positive results of overall catheter tip culture (n = 171 for ethanol; n = 176 for placebo).

Neither treatment with TPN nor stay at the intensive care unit, underlying disease, neutropenia at time of catheter placement, catheter insertion site, or number of catheter lumens contributed individually to the development of CRBSI (data not shown).

### Secondary goals

Results are presented in Table 1 and 2. The mean catheter dwell time was 63.1 days (range, 2–486 days) for ethanol locks versus 60.7 days (range, 4–308 days) for placebo (P = .71). The mean duration of the use of a glycopeptides (6.0 versus 5.0 days) did not differ between patients randomized to ethanol locks or placebo (P = .62). Also, the duration of treatment with other classes of systemic antibiotics did not differ between ethanol locks (mean duration, 17.4 days), or placebo (mean duration, 16.7 days). Overall CRBSI was recorded in 21 of 226 patients allocated to ethanol locks versus 24 of 222 patients treated with placebo (P = .71). For overall bacteremia, these results were 91 of 226 versus 91 of 222 patients, respectively (P = .95).

Tip cultures were performed on 347 catheters. Rates of detection of microbial growth were 49 of 171 in patients treated with ethanol locks and 57 of 176 in patients allocated to placebo (P = .52). Of all catheter episodes in which bacteremia was documented (n = 182), CNS was detected as the causative pathogen in 106 episodes (58%), which was equally distributed between both groups, as were other causing microbes (Table 3). Hub cultures were performed in 147 patients, and were positive in 8 patients in the ethanol arm versus 11 patients in the placebo arm (P = .67). However, different strains were obtained from hubs as compared with blood cultures in 4 patients, (n = 2 for ethanol and placebo). Therefore, not all patients with positive hub cultures qualified for endoluminal CRBSI.

**Table 3:** Overview of cultured microbes in case of bacteremia (182 episodes).

	Ethanol	Placebo
	(n = 91)	(n = 91)
CNSa,b	49	57
Other skin colonizers	2	2
*Staphylococcus aureus*	2	3
Other gram-positive cocci	12	10
Gram-negatives	4	5
Polymicrobial	20	13
Yeasts	2	1

Data represent numbers of episodes of bacteremia. CNS, coagulase-negative staphylococci.

a Of all 106 episodes with CNS-bacteremia for which glycopeptide-therapy was started, 32 were due to CRBSI (including endoluminal and exoluminal infection). Of the remaining 74 episodes, tentative sources were mucositis (n = 21), cytarabin skin toxicity (n = 8), contaminated blood cultures (n = 8), red catheter insertion site without other criteria for exoluminal CRBSI (n = 6), unknown (n = 29), and other causes (n = 2).

### Safety and tolerability aspects

Data are presented in Table 4. All-cause mortality in patients allocated to ethanol locks was 7 of 226, compared with 5 of 222 patients randomized to placebo (P = .77). None of the involved deaths were diagnosed with CRBSI during the catheter episode.

**Table 4:** Tolerability and safety of study compound.

			Ethanol	Placebo	P
**Total cohort**			(n = 226)	(n = 222)	
	All-cause mortality		7	5	.77
	Thrombosis of insertion blood vessel		9	12	.62
	Discontinuation of study compound		10	0	.002*
		Modified lock frequency	11	1	.006*
		Complete cessation	2	9	.50
	Other events^a^				
**Questionnaire** b			(n = 88)	(n = 93)	
	Subjective parameters				
		Facial flushing	39	17	<.001*
		Nausea/vomiting	20	17	.58
		Altered taste	31	19	.04*
		Feelings of dizziness/drowsiness	41	10	<.001*

Data represent numbers of events; *denotes statistical significance.

a One patient had syncope right after flushing the first lock solution into the circulation, 1 device had to be removed because of a rupture of a catheter lumen which occurred during sleep.

b The predefined analysis of subjective adverse effects was performed on a random sample of the total cohort by means of a questionnaire.

No differences were observed in the incidence of thrombosis. In patients allocated to ethanol locks, 1 device had to be removed because of a rupture of 1 of the 3 catheter lumens, which occurred while the patient was asleep. No life-threatening adverse events were observed. One ethanol treated patient had syncope shortly after flushing through the first lock solution. During subsequent ethanol lock procedures, no further adverse effects occurred in this particular patient. Significantly more patients receiving ethanol locks discontinued lock therapy (P = .006) or continued with a frequency-adjusted regimen (P = .002), as compared to placebo. This was due to subjective feelings of discomfort, including facial redness or flushing, feelings of drowsiness or an alcohol taste after flushing the lock solution through. No differences in levels of hepatic enzymes (aspartate-aminotransferase, g-glutamyl transpeptidase) and mean corpuscular volume of red blood cells were observed after 2 weeks of lock therapy when compared to baseline values (P>.5 for all parameters; data not shown).

## Discussion

The present randomized clinical trial on the use of a preventive ethanol lock showed a non-significant 41%-reduction of endoluminal CRBSI in patients allocated to ethanol locks for occurrence of CRBSI as expressed per 1000 CVC-days. Also, the 3.6-fold reduction as observed in ethanol lock patients who classified for endoluminal CRBSI according to the strictest definition was not significant. No differences were observed for catheter dwell time, use of glycopeptides and other systemic antibiotics, and rates of overall CRBSI or bacteremia between groups.

In patients treated with ethanol locks, 1 device had to be removed due to loss of integrity of the CVC; another person experienced an episode of syncope after the first lock procedure but not after subsequent procedures. No other serious adverse events were observed, which is in agreement with other reported data.[20] Significantly more patients treated with ethanol locks discontinued their prophylactic treatment. All reported adverse effects were non-severe but reasonably ethanol related. In future studies, this may partially be circumvented by removing the lock solution instead of flushing it through, as has safely been done in other recent studies.[21], [29]

We took efforts to perform a double-blind, randomized trial. However, due to the specific ethanol odour that could be sensed after opening the ampoules by the nursing team, blinding was not 100% in daily practice. Nevertheless, the principal investigators were not directly involved in patient management and were therefore completely blinded at all time. Furthermore, the primary endpoint has no subjective element in its definition, which may reasonably minimize potential bias.

Currently, several promising observational in vivo data on the *treatment* of CRBSI with ethanol locks have been reported.[15]–[17] Overall tolerance of ethanol was good in these studies and no significant adverse effects were observed. Furthermore, several case-series on the use of *preventive* ethanol locks have been published. In a recent case-series, Mouw and colleagues described 10 TPN-dependent paediatric patients with tunnelled catheters, who were treated with a 70%-ethanol lock solution between TPN infusions.[19] Infection rates in 5 children of whom data were available from the period before initiation of lock therapy declined from 11.2 to 2.1 CRBSIs per 1000 CVC-days. In a recent small randomized trial, Sanders and colleagues observed a reduced incidence of CRBSI with a 70%-ethanol lock in hematology patients with tunnelled CVCs.[21] CRBSI occurred in 3 versus 11 patients in the ethanol and control groups, respectively (odds-ratio, 0.18; 95% confidence interval, 0.05–0.65). Catheter survival was longer in the ethanol group (P = .003). Several differences with our study should be taken into account. First, Sanders et al. used less stringent CRBSI definitions. With this respect, it is surprising that the large majority of CRBSIs was caused by gram-negative microorganisms instead of staphylococci. One wonders whether these episodes of bacteremia were the consequence of translocation from the gut rather than CRBSI. The lack of stringent definitions may also partly explain the high incidence of CRBSI (31 per 1000 CVC-days) in the control group, which is around 16 times lower in our present study (1.19 per 1000 CVC-days) and another landmark study.[28] Interestingly, the preliminary data of a randomized trial performed by Crnich and colleagues, including 359 long-term tunnelled or implanted CVCs, showed no benefit of the use of a 50%-ethanol lock for CRBSI-prevention in hospitalized patients.[29]

Ethanol acts bactericidal and fungicidal against a broad range of bacteria and even yeasts without concerns of resistance development.[18] In vitro, it has been demonstrated that a 15%-ethanol concentration was able to kill most planktonic microorganisms.[30] For microorganisms in established biofilms, which is the case in CVCs, concentrations of 40% to 70% were required to achieve a bactericidal effect because penetration into a biofilm is harder to establish.[31] As a concern, it has been reported that a 100%-ethanol lock solution was associated with catheter occlusion.[32] Another report showed that infusion of polyurethane catheters with 70%-ethanol resulted in qualitative softening of the catheters.[33] More recently, however, no changes were observed on the biomechanical properties of polyurethane catheters, which were submerged in an ethanol solution for 9 weeks.[34] An overview of all recent studies, discussing the most relevant aspects of the ethanol lock technique was published recently.[35]

Several factors may explain the lack of efficacy as observed in our study. First, for practical reasons we used a lock time of 15 minutes daily per catheter lumen. This was decided because a longer dwell time would have interfered too much with patient care. A recent in vitro study showed that a significant 3-log reduction in the number of biofilm-associated gram-positive cocci occurred already after 20 minutes exposure to a 60%-ethanol lock solution. A dwell time of 30 minutes was required for complete eradication.[36] However, another in vitro study showed recently that an exposure time of 1 minute to a 70%-ethanol solution was sufficient for the sterilization of a bacterial biofilm.[37]

Second, a lock-based intervention will reasonably prevent only *endoluminal* CRBSI. By employing strict definitions we tried to differentiate endoluminal CRBSI from other entities. However, the true sensitivity of hub cultures to detect endoluminal infection is unknown. Furthermore, the incidence of strictly endoluminal CRBSI in the placebo arm was as low as 0.032% (7 of 222 patients). Therefore, the study was underpowered in retrospect. Taking this incidence rate into account, it can be calculated that future studies in comparable patient populations should include 848 patients to demonstrate a 75% reduction with 80% power, which is even augmented to 2282 patients to be able to show a 50% reduction of strictly endoluminal CRBSI.

Finally, bacteremia with CNS is not always CVC-related, but may result from translocation from the bowel in patients with severe mucositis.[38], [39] This could explain why despite the high overall incidence of CNS bacteremia, which occurred in 106 of all 182 episodes of bacteremia, no reduction of CNS bacteremia was seen due to the use of ethanol locks. The use of antimicrobial prophylaxis resulting in selective eradication of intestinal gram-negative but not gram-positive microorganisms may be the underlying cause. To test this hypothesis, we did genotypic identification of CNS in a random sample of 15 patients with documented bacteremia who were found to have concomitant CNS in rectal and/or vaginal mucosa or mouth swabs. Identical CNS strains in blood and mucosa were identified in 6 of 15 patients (40%).

Although the relevance of mucositis-associated bacteremia is not fully elucidated yet, it may be hypothesized that an intervention with an endoluminal CVC lock will not result in a reduction of *overall* bacteremia in patients who are treated with high-dose chemotherapy, and nearly inevitably suffer from severe mucositis. Also, the observed rate of exoluminal compared to endoluminal CRBSI in our study was higher than expected. Both these aspects may explain why our initial hypothesis that the rate of CNS bacteremia as observed in other studies reflects mainly *endoluminal* CRBSI may have been inaccurate in retrospect. In this view, the observed 3.6-fold reduction of strictly endoluminal CRBSI in patients allocated to ethanol locks is reassuring, as is the 41%-reduction of endoluminal CRBSI as expressed per 1000 CVC-days, because the lack of statistical significance may reflect a lack of power more than a lack of effectiveness.

However, the overall incidence of endoluminal CRBSI in our patients was low. One wonders whether the clinical benefits of this intervention, even in case of a significant reduction of endoluminal CRBSI, in this specific patient population would outweigh the extra amount of effort, costs and patient discomfort. Additional studies should therefore be performed in populations with higher incidence of (endoluminal) CRBSI, e.g., patients receiving long-term treatment with TPN.

## Supporting Information

Checklist S1 CONSORT checklist
(0.19 MB DOC)

Protocol S1 Trial Protocol
(0.16 MB DOC)
