# Supplementary material for: Prevention of Catheter-Related Bacteremia with a Daily Ethanol Lock in Patients with Tunnelled Catheters: A Randomized, Placebo-Controlled Trial
Source: PLoS One. 2010 May 26;5(5):e10840. doi: 10.1371/journal.pone.0010840 (PMC2877107; doi:10.1371/journal.pone.0010840)
Supplement: Protocol S1 — Trial Protocol (0.16 MB DOC) [file pone.0010840.s002.doc]

**AlcLock protocol version 2.9.**

Prevention of catheter-related infections with a daily alcohol Lock. Randomised placebo controlled trial.

BJ Rijnders, L Slobbe, PJ Lugtenburg, S de Marie, MC Vos, JK Doorduijn.

29/08/2026

[*Introduction and background information* 1](#__RefHeading___Toc67147976)

[*Methods* 2](#__RefHeading___Toc67147977)

[*The following inclusion criteria have to be fulfilled* 2](#__RefHeading___Toc67147978)

[*The following exclusion criteria have to be considered before inclusion* 2](#__RefHeading___Toc67147979)

[*Preparation and use of alcohol lock* 3](#__RefHeading___Toc67147980)

[*The primary and secondary endpoints* 3](#__RefHeading___Toc67147981)

[*Randomization, allocation concealment and blinding* 4](#__RefHeading___Toc67147982)

[*Data and clinical sample collection and follow-up* 5](#__RefHeading___Toc67147983)

[*Sample size considerations* 6](#__RefHeading___Toc67147984)

[*Statistical analysis:* 6](#__RefHeading___Toc67147985)

[Addendum 1 9](#__RefHeading___Toc67147986)

[Addendum 2 10](#__RefHeading___Toc67147987)

[Addendum 3 13](#__RefHeading___Toc67147988)

# *Introduction and background information*

In modern-day medicine, the use of intravascular catheters has become unavoidable. In the United States, hospitals and clinics purchase more than 150 million intravascular devices each year of which more than 5 million are central venous catheters(1). However, their use does not come without risk. It puts patients at danger for mechanical, thrombotic and infectious complications (catheter-related infections, CRI), the most important of which is catheter-related *bloodstream* infection (CRBSI). CRBSI is associated with significant increase in hospital stay and therefore cost of patient management, morbidity and probably also mortality(2-4). The increase in expenses was estimated to be 15.965 US dollars per patient with a CRBSI in 1994 and even 56167 US dollars in another more recent study(5).

It is clear that the prevention of CRI is of utmost importance and will help to decrease patient suffering as well as cost of patient management. Extensive and detailed evidence-based recommendations for the prevention of CRI were recently published(6). However, many topics remain unresolved and there still is an urgent need for effective, cheap and easy to implement preventive measures that are without risk of developing antibiotic resistance.

Catheters can become colonized with microorganisms through exoluminal (catheter insertion site) or endoluminal (hub and infusates) routes. It has been shown that, the longer a catheter remains in place, the more important the endoluminal route becomes(7). The endoluminal route of infection can be prevented to some extent when an *antibiotic* solution is instilled in the catheter for a long enough time and on a regular basis(8-11). However, to avoid resistance from occurring, the use of antibiotics in such a lock for infection prevention should remain exceptional. Although there is evidence to support the concept(12;13), methodologically appropriate clinical data on the use of *antiseptic* solutions for this purpose are still awaited.

Recently, the use of an alcohol lock solution was suggested as a promising way to prevent CRBSI(14) and the compatibility of polyurethane and silicone catheters submerged in an alcohol solution was publicized with no biomechanical or structural changes detected after 9 weeks of immersion(15). The major advantage of an alcohol lock solution would be the broad antimicrobial spectrum without the risk of compromising future antibiotic treatment as, in contrast to the use of an antibiotic lock, the development of antibiotic resistance is not of concern. Furthermore it would be cheap and universally available.

In this randomised study the efficacy of a 70% alcohol lock solution for the prevention of CRBSI will be compared with placebo when applied for 30 minutes per day.

# *Methods*

### *The following inclusion criteria have to be fulfilled*

- Patient at least 18 years old
- Admitted to the haematology department
- With a tunnelled central venous catheter inserted in the preceding 72hrs.

### *The following exclusion criteria have to be considered before inclusion*

- Known allergy to alcohol or active use of metronidazol (or related 2-nitroimidazole compounds) or Disulfiram (Antabuse)

### *Preparation and use of alcohol lock*

The ethyl alcohol solution that will be used to lock the catheter is a 70% aqueous solution. The hospital pharmacist delivers the alcohol and placebo locks to the medical ward in single use syringes of 5cc. During hospitalisation, every lumen of the central venous catheter is locked with an alcohol lock solution or matching placebo (0,9% NaCl) for 30 minutes per day. After discharge, the lock will be instilled once weekly for 30 minutes. This is at the time the patient visits the hospital to have the heparin solution (with which the catheter is continuously filled) renewed.

### *The primary and secondary endpoints*

#### Primary endpoint

The primary endpoint is catheter related bacteremia originating at the catheter hub. It is a composite endpoint existing of ***any*** of the following;

- Any bacteremia for which the catheter was removed and with all of the following findings;
  1. A positive hub culture
  2. A positive catheter tip culture
  3. A positive blood culture. Only one positive blood culture was required when a peripheral blood culture was available but 2 positive blood cultures were required when drawn through the catheter.
  4. Same genotype of the strain from the hub, tip and blood culture
- Any bacteremia with a *DTTP >2hrs* and with a positive hub blood culture with the same genotypic strain as in the blood. DTTP denotes difference in time to positivity of a blood culture taken through a new puncture site minus the time to positivity of a blood culture taken through the catheter. A DTTP > 2hrs accurately predicts the catheter to be the source of a bacteremia*(16;17).*

A bacteremia with DTTP >2hrs and with the same genotypic strain cultured in blood and from the insertion site but with a negative hub culture was *not* considered a primary endpoint as an alcohol lock cannot be expected to prevent a catheter-related infection originating at the insertion site.

If at the time of catheter removal (because no longer needed) or death of the patient no primary endpoint is diagnosed, the treatment is considered successful.

##### Secundary endpoint

1. Bacteremia with a DTTP>2 hrs but not fulfilling the primary endpoint criteria (no positive hub culture)
2. Catheter survival time (=premature catheter removal)
3. Vancomycin use in days that vancomycin (any dose) was administered.
4. Incidence of positive catheter tip cultures(18)
5. Incidence of overall bacteremia/fungemia for which antibiotic treatment was given (Gram-positive, negative and yeasts)

### *Randomization, allocation concealment and blinding*

**Randomisation and allocation concealment;** The allocation sequence is located at the clinical pharmacy of the hospital during the duration of the trial and only the pharmacists have access to it. Pharmacists, of whom nobody is co-investigator of the study, perform patient randomisation. Computer generated random number tables are used. Randomisation will be done in blocks of 8 without stratification.

**Blinding;** Alcohol 70% has a specific odor. Therefore to be able to use a matching placebo, 5ml syringes were filled with alcohol or placebo (0.9% NaCl) and individually packed in a sealed plastic bags. Inside these bags was put a drop of Lavender oil to conceal the odor of alcohol.

### *Data and clinical sample collection and follow-up*

As the inclusion document (*addendum 1* to this protocol) shows, the following base-line data will be collected at the time of inclusion: Age, underlying disease, date of catheter insertion, type of catheter, neutrophil count and antibiotic use during the previous days.

As the follow-up document shows (*addendum 2*), at the time a primary endpoint is diagnosed, hub and insertion site cultures will be performed. DTTP or quantitative blood culture ratios of the peripheral blood cultures versus the blood cultures taken through the catheter are registered whenever available. For this purpose, the treating physicians and nurses of the haematology departments will be constantly urged to always take a peripheral AND through-the-catheter blood culture for any *new* episode of fever (38,3°C) or sepsis.

Patients are followed until a primary endpoint arises, the catheter is withdrawn or the patient dies (whichever comes first). The exit site is cultured whenever inflammation is noted at the insertion site or bacteremia without a clinical focus is diagnosed. For the latter a hub culture is performed as well.

Strain identification and time to positivity analysis of blood cultures are performed with the VITEC 2 system and with the use of routine microbiological methods. All catheters are cultured when removed at all times and reason for catheter removal is noted. Catheter-tip culture is performed using the sonication method with a cut-off of 100 cfu/catheter(19) and the roll-plate method with a cut-off of 15 cfu/catheter(20).

Genotyping will be performed as previously described. In brief a suspension of bacteria is mixed with 1% InCert agarose (FMC Bioproducts, Rockland, ME, USA). Agarose plugs are incubated with lysostaphin (Sigma Chemicals, St. Louis, MO, USA). Spheroplasts are lysed and plugs are washed in 10 mmol l-1 Tris–HCl pH 8.0 and 1 mmol l-1 EDTA and stored at 4°C. DNA is digested by SmaI (Boehringer-Mannheim, Mannheim, Germany), and PFGE carried out in 1% SeaKem GTG agarose gels (FMC Bioproducts). The buffer consists of 0.5×TBE; the temperature is set at 14°C in a Bio-Rad CHEF Mapper (Bio-Rad, Veenendaal, The Netherlands). Running time is 22 h, with linear ramping from 2.16 to 44.69 s at an angle 120° (60°/-60°). The voltage is 6 V cm-1. Gels are stained with ethidium bromide and photographed. Differences in banding patterns are documented by two independent observers. Genotypes are defined on the basis of identity of the DNA banding patterns. Subtypes differed in the position of one or two restriction fragments.

### *Sample size considerations*

For the primary endpoint

As the primary endpoint can be expected to occur in 10% of the study population (21) and a 80% reduction with the use of an alcohol lock is anticipated, we calculated that 135 patients are needed in each group to detect a (2-sided) difference with 80% power and alfa=0.05.

**For the secundary endpoint**

As the most important secondary endpoint (catheter-related bacteremia defined as bacteremia with a DTTP >2 hrs) can be expected to occur in 20% of the study population (21) and a 50% reduction with the use of an alcohol lock is anticipated, we calculated that 219 patients are needed in each group to detect a (2-sided) difference with 80% power and alfa=0.05.

###

### *Statistical analysis:*

Analysis will be done following the intention-to-treat principles, including all randomised patients that received at least one Alcohol-Lock (modified intention-to-treat).

The primary endpoint analysis will consist of the comparison of time to treatment failure (the primary endpoint) between placebo and alcohol‑locks at time of catheter-removal or dead of the patient (whichever comes first) using logrank test at 0.05 (two‑sided) significance level.

The secondary endpoint analysis will consist of the comparison of time to the secondary endpoints between controls and alcohol‑lock patients at the time of catheter-removal or dead of the patient (whichever comes first) for the first 2 secondary endpoints. For secundary endpoints 3, 4 and 5 proportions will be compared between groups.

*Ethical issues:*

Written informed consent is needed from all patients (addendum 3)

Reference List

(1) Mermel LA, Farr BM, Sherertz RJ, Raad II, O'Grady N, Harris JS et al. Guidelines for the management of intravascular catheter-related infections. Clin Infect Dis 2001; 32(9):1249-1272.

(2) Digiovine B, Chenoweth C, Watts C, Higgins M. The attributable mortality and costs of primary nosocomial bloodstream infections in the intensive care unit. Am J Respir Crit Care Med 1999; 160(3):976-981.

(3) Rello J, Ochagavia A, Sabanes E, Roque M, Mariscal D, Reynaga E et al. Evaluation of outcome of intravenous catheter-related infections in critically ill patients. Am J Respir Crit Care Med 2000; 162(3 Pt 1):1027-1030.

(4) Renaud B, Brun-Buisson C. Outcomes of primary and catheter-related bacteremia. A cohort and case-control study in critically ill patients. Am J Respir Crit Care Med 2001; 163(7):1584-1590.

(5) Dimick JB, Pelz RK, Consunji R, Swoboda SM, Hendrix CW, Lipsett PA. Increased resource use associated with catheter-related bloodstream infection in the surgical intensive care unit. Arch Surg 2001; 136(2):229-234.

(6) O'Grady NP, Alexander M, Dellinger EP, Gerberding JL, Heard SO, Maki DG et al. Guidelines for the prevention of intravascular catheter-related infections. Centers for Disease Control and Prevention. MMWR Recomm Rep 2002; 51(RR-10):1-29.

(7) Raad I, Costerton W, Sabharwal U, Sacilowski M, Anaissie E, Bodey GP. Ultrastructural analysis of indwelling vascular catheters: a quantitative relationship between luminal colonization and duration of placement. J Infect Dis 1993; 168(2):400-407.

(8) Carratala J, Niubo J, Fernandez-Sevilla A, Juve E, Castellsague X, Berlanga J et al. Randomized, double-blind trial of an antibiotic-lock technique for prevention of gram-positive central venous catheter-related infection in neutropenic patients with cancer. Antimicrob Agents Chemother 1999; 43(9):2200-2204.

(9) Schwartz C, Henrickson KJ, Roghmann K, Powell K. Prevention of bacteremia attributed to luminal colonization of tunneled central venous catheters with vancomycin-susceptible organisms. J Clin Oncol 1990; 8(9):1591-1597.

(10) Dogra GK, Herson H, Hutchison B, Irish AB, Heath CH, Golledge C et al. Prevention of tunneled hemodialysis catheter-related infections using catheter-restricted filling with gentamicin and citrate: a randomized controlled study. J Am Soc Nephrol 2002; 13(8):2133-2139.

(11) Henrickson KJ, Axtell RA, Hoover SM, Kuhn SM, Pritchett J, Kehl SC et al. Prevention of central venous catheter-related infections and thrombotic events in immunocompromised children by the use of vancomycin/ciprofloxacin/heparin flush solution: A randomized, multicenter, double-blind trial. J Clin Oncol 2000; 18(6):1269-1278.

(12) Chatzinikolaou I, Zipf TF, Hanna H, Umphrey J, Roberts WM, Sherertz R et al. Minocycline-ethylenediaminetetraacetate lock solution for the prevention of implantable port infections in children with cancer. Clin Infect Dis 2003; 36(1):116-119.

(13) Allon M. Prophylaxis against dialysis catheter-related bacteremia with a novel antimicrobial lock solution. Clin Infect Dis 2003; 36(12):1539-1544.

(14) Maki DG, Crnich CJ, Safdar N. Successful use of a 25% Alcohol Lock Solution for Prevention of Recurrent CVC-Related Bloodstream Infection in a patient on Home TNA. 42nd ICAAC Abstracts, American Society for Microbiology, September 27 - 30, 2002, San Diego, CA, page 320 . 2002.

(15) A.Aiyangar, W.C.Crone, C.J.Crnich DGM. Effect of Ethanol on the Mechanical Properties of Polyurethane Catheters. Proceedings of the 2002 SEM Annual Conference and Exposition on Experimental and Applied Mechanics . 2002.

(16) Blot F, Nitenberg G, Chachaty E, Raynard G, Germann N, Antoun S et al. Diagnosis of catheter-related bacteraemia: a prospective comparison of the time to positivity of hub-blood versus peripheral-blood cultures. Lancet 1999; 354 (9184):1071-1077.

(17) Seifert H, Cornely O, Seggewiss K, Decker M, Stefanik D, Wisplinghoff H et al. Bloodstream infection in neutropenic cancer patients related to short- term nontunnelled catheters determined by quantitative blood cultures, differential time to positivity, and molecular epidemiological typing with pulsed-field gel electrophoresis. J Clin Microbiol 2003; 41(1):118-123.

(18) Rijnders BJ, Van Wijngaerden E, Peetermans WE. Catheter-tip colonization as a surrogate end point in clinical studies on catheter-related bloodstream infection: how strong is the evidence? Clin Infect Dis 2002; 35(9):1053-1058.

(19) Sherertz RJ, Raad II, Belani A, Koo LC, Rand KH, Pickett DL et al. Three-year experience with sonicated vascular catheter cultures in a clinical microbiology laboratory. J Clin Microbiol 1990; 28(1):76-82.

(20) Maki DG, Weise CE, Sarafin HW. A semiquantitative culture method for identifying intravenous-catheter-related infection. N Engl J Med 1977; 296(23):1305-1309.

(21) Nouwen JL, Wielenga JJ, van Overhagen H, Lameris JS, Kluytmans JA, Behrendt MD et al. Hickman catheter-related infections in neutropenic patients: insertion in the operating theater versus insertion in the radiology suite. J Clin Oncol 1999; 17(4):1304.

# Addendum 1

# Inclusion form Alcohol-Lock study

PATIENT ID

Nr.

**Date of inclusion** (dd/mm/yyyy): |__|__| |__|__| |__|__|__|__|

**Inclusion criteria that need to be fulfilled**

Patient at least 18 years old

Tunnelled central venous catheter inserted in the preceding 72hrs

**Exclusion criteria**

Known allergy to alcohol

Concomitant use of metronidazol, tinidazol or disulfiram

**Baseline characteristics:**

1. Catheter type: ……………………….. Single / double / triple lumen
2. Date of insertion of the catheter (dd/mm/yyyy): |__|__| |__|__| |__|__|__|__|
3. Neutrophil count at time of insertion |__|__|.|__| x 10^9^ cells/L
4. Underlying disease for which the catheter was inserted? .................................................
5. SDD at the time of catheter insertion ? Yes No

If yes, which one ? Colistin / Ciproxin / Tobramycin /

Diflucan / Amfotericin

1. Antibiotics at the time of insertion ? Yes No

If yes, which one ? ..................................................

If yes since when (dd/mm/yyyy): |__|__| |__|__| |__|__|__|__|

**Full name and signature** .................................................

# Addendum 2

#### Patient ID

# Follow-up AB-Lock studie

Nr.

Deze patient maakt deel uit van de AlcLock studie en krijgt dus Alcohol (of

placebo) als preventie van catheterinfectie.

Wanneer er een nieuw vermoeden van catheterinfectie is gelieve dan de

behandelend arts (dienstdoende arts) dit papier te laten lezen.

**Alvorens vancomycine te starten dienen er vaak vooraf nog een aantal kweken afgenomen te worden (met name bloedkweek via de catheter en via perifere punctie, kweek van de catheter-hub, kweek van de insteekplaats).**

U kan hierover zo nodig verder informatie inwinnen via :

1. bieper *35339 (via ErasmusMCcentrumlocatie tel. 010.4639222)
2. Na 17u en in het weekend: Dienstdoende microbioloog (via 010.4639222)
3. Indien 1 en 2 niet lukt via 0032497510077

Het gebruik van de studiespuitjes gebeurt als volgt;

1. Het lumen dat zal worden gevuld met de studieoplossing wordt eerst doorgespoeld met 10cc fysiologische zoutoplossing (NaCl 0.9%).
2. Vervolgens wordt het lumen gevuld met de studieoplossing en verder niet meer gebruikt gedurende 30 minuten.
3. Na deze 30 minuten wordt de studieoplossing ***DOORGESPOELD*** met 10 cc fysiologische zoutoplossing (NaCL 0.9%).
4. Doorstreep tenslotte op de achterkant van dit blad op de datum van vandaag het lumen dat je afgesloten had.

**Bij vragen of twijfel tel. bieper *35339 via tel. Centrale van ErasmusMC centrumlocatie 010.463922 (na 17u00 of indien afwezig: 0032.497.510077)**

***Wanneer de catheter wordt verwijderd dit papier meegeven aan de infectioloog (patientenbespreking op dinsdag of donderdagvoormiddag) of opsturen naar;***

***dr. BJA Rijnders, ERASMUS MC centrumlocatie. Kamer D418.***

|  |  |  |  |  |  |  |  |  |  |  |  |  |  |
| --- | --- | --- | --- | --- | --- | --- | --- | --- | --- | --- | --- | --- | --- |
| **28/01/04** | **29/01/04** | **30/01/04** | **31/01/04** | **01/02/04** | **02/02/04** | **03/02/04** | **04/02/04** | **05/02/04** | **06/02/04** | **07/02/04** | **08/02/04** | **09/02/04** | **10/02/04** |
| Lumen 1 | Lumen 1 | Lumen 1 | Lumen 1 | Lumen 1 | Lumen 1 | Lumen 1 | Lumen 1 | Lumen 1 | Lumen 1 | Lumen 1 | Lumen 1 | Lumen 1 | Lumen 1 |
| Lumen 2 | Lumen 2 | Lumen 2 | Lumen 2 | Lumen 2 | Lumen 2 | Lumen 2 | Lumen 2 | Lumen 2 | Lumen 2 | Lumen 2 | Lumen 2 | Lumen 2 | Lumen 2 |
| Lumen 3 | Lumen 3 | Lumen 3 | Lumen 3 | Lumen 3 | Lumen 3 | Lumen 3 | Lumen 3 | Lumen 3 | Lumen 3 | Lumen 3 | Lumen 3 | Lumen 3 | Lumen 3 |
| **11/02/04** | **12/02/04** | **13/02/04** | **14/02/04** | **15/02/04** | **16/02/04** | **17/02/04** | **18/02/04** | **19/02/04** | **20/02/04** | **21/02/04** | **22/02/04** | **23/02/04** | **24/02/04** |
| Lumen 1 | Lumen 1 | Lumen 1 | Lumen 1 | Lumen 1 | Lumen 1 | Lumen 1 | Lumen 1 | Lumen 1 | Lumen 1 | Lumen 1 | Lumen 1 | Lumen 1 | Lumen 1 |
| Lumen 2 | Lumen 2 | Lumen 2 | Lumen 2 | Lumen 2 | Lumen 2 | Lumen 2 | Lumen 2 | Lumen 2 | Lumen 2 | Lumen 2 | Lumen 2 | Lumen 2 | Lumen 2 |
| Lumen 3 | Lumen 3 | Lumen 3 | Lumen 3 | Lumen 3 | Lumen 3 | Lumen 3 | Lumen 3 | Lumen 3 | Lumen 3 | Lumen 3 | Lumen 3 | Lumen 3 | Lumen 3 |
| **25/02/04** | **26/02/04** | **27/02/04** | **28/02/04** | **29/02/04** | **01/03/04** | **02/03/04** | **03/03/04** | **04/03/04** | **05/03/04** | **06/03/04** | **07/03/04** | **08/03/04** | **09/03/04** |
| Lumen 1 | Lumen 1 | Lumen 1 | Lumen 1 | Lumen 1 | Lumen 1 | Lumen 1 | Lumen 1 | Lumen 1 | Lumen 1 | Lumen 1 | Lumen 1 | Lumen 1 | Lumen 1 |
| Lumen 2 | Lumen 2 | Lumen 2 | Lumen 2 | Lumen 2 | Lumen 2 | Lumen 2 | Lumen 2 | Lumen 2 | Lumen 2 | Lumen 2 | Lumen 2 | Lumen 2 | Lumen 2 |
| Lumen 3 | Lumen 3 | Lumen 3 | Lumen 3 | Lumen 3 | Lumen 3 | Lumen 3 | Lumen 3 | Lumen 3 | Lumen 3 | Lumen 3 | Lumen 3 | Lumen 3 | Lumen 3 |
| **10/03/04** | **11/03/04** | **12/03/04** | **13/03/04** | **14/03/04** | **15/03/04** | **16/03/04** | **17/03/04** | **18/03/04** | **19/03/04** | **20/03/04** | **21/03/04** | **22/03/04** | **23/03/04** |
| Lumen 1 | Lumen 1 | Lumen 1 | Lumen 1 | Lumen 1 | Lumen 1 | Lumen 1 | Lumen 1 | Lumen 1 | Lumen 1 | Lumen 1 | Lumen 1 | Lumen 1 | Lumen 1 |
| Lumen 2 | Lumen 2 | Lumen 2 | Lumen 2 | Lumen 2 | Lumen 2 | Lumen 2 | Lumen 2 | Lumen 2 | Lumen 2 | Lumen 2 | Lumen 2 | Lumen 2 | Lumen 2 |
| Lumen 3 | Lumen 3 | Lumen 3 | Lumen 3 | Lumen 3 | Lumen 3 | Lumen 3 | Lumen 3 | Lumen 3 | Lumen 3 | Lumen 3 | Lumen 3 | Lumen 3 | Lumen 3 |
| **24/03/04** | **25/03/04** | **26/03/04** | **27/03/04** | **28/03/04** | **29/03/04** | **30/03/04** | **31/03/04** | **01/04/04** | **02/04/04** | **03/04/04** | **04/04/04** | **05/04/04** | **06/04/04** |
| Lumen 1 | Lumen 1 | Lumen 1 | Lumen 1 | Lumen 1 | Lumen 1 | Lumen 1 | Lumen 1 | Lumen 1 | Lumen 1 | Lumen 1 | Lumen 1 | Lumen 1 | Lumen 1 |
| Lumen 2 | Lumen 2 | Lumen 2 | Lumen 2 | Lumen 2 | Lumen 2 | Lumen 2 | Lumen 2 | Lumen 2 | Lumen 2 | Lumen 2 | Lumen 2 | Lumen 2 | Lumen 2 |
| Lumen 3 | Lumen 3 | Lumen 3 | Lumen 3 | Lumen 3 | Lumen 3 | Lumen 3 | Lumen 3 | Lumen 3 | Lumen 3 | Lumen 3 | Lumen 3 | Lumen 3 | Lumen 3 |
| **07/04/04** | **08/04/04** | **09/04/04** | **10/04/04** | **11/04/04** | **12/04/04** | **13/04/04** | **14/04/04** | **15/04/04** | **16/04/04** | **17/04/04** | **18/04/04** | **19/04/04** | **20/04/04** |
| Lumen 1 | Lumen 1 | Lumen 1 | Lumen 1 | Lumen 1 | Lumen 1 | Lumen 1 | Lumen 1 | Lumen 1 | Lumen 1 | Lumen 1 | Lumen 1 | Lumen 1 | Lumen 1 |
| Lumen 2 | Lumen 2 | Lumen 2 | Lumen 2 | Lumen 2 | Lumen 2 | Lumen 2 | Lumen 2 | Lumen 2 | Lumen 2 | Lumen 2 | Lumen 2 | Lumen 2 | Lumen 2 |
| Lumen 3 | Lumen 3 | Lumen 3 | Lumen 3 | Lumen 3 | Lumen 3 | Lumen 3 | Lumen 3 | Lumen 3 | Lumen 3 | Lumen 3 | Lumen 3 | Lumen 3 | Lumen 3 |
| **21/04/04** | **22/04/04** | **23/04/04** | **24/04/04** | **25/04/04** | **26/04/04** | **27/04/04** | **28/04/04** | **29/04/04** | **30/04/04** | **01/05/04** | **02/05/04** | **03/05/04** | **04/05/04** |
| Lumen 1 | Lumen 1 | Lumen 1 | Lumen 1 | Lumen 1 | Lumen 1 | Lumen 1 | Lumen 1 | Lumen 1 | Lumen 1 | Lumen 1 | Lumen 1 | Lumen 1 | Lumen 1 |
| Lumen 2 | Lumen 2 | Lumen 2 | Lumen 2 | Lumen 2 | Lumen 2 | Lumen 2 | Lumen 2 | Lumen 2 | Lumen 2 | Lumen 2 | Lumen 2 | Lumen 2 | Lumen 2 |
| Lumen 3 | Lumen 3 | Lumen 3 | Lumen 3 | Lumen 3 | Lumen 3 | Lumen 3 | Lumen 3 | Lumen 3 | Lumen 3 | Lumen 3 | Lumen 3 | Lumen 3 | Lumen 3 |

# Addendum 3


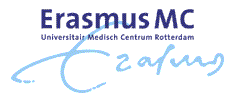


INFORMED CONSENT formulier van de ***Alcohol-Lock Study***

*Inleiding:*

Recent werd bij u een catheter in de bloedbaan geplaatst (Hickmann catheter). Dit omdat de behandeling die u nodig heeft alleen via het gebruik van zo’n catheter mogelijk is. In dit verband informeerde uw behandeld arts u eveneens over de alcohol-Lock studie die momenteel wordt uitgevoerd in het Erasmus Medisch Centrum (centrumlocatie en lokatie Daniel den Hoed). Hij/zij heeft u al het een en ander uitgelegd. Voor toestemming of weigering is goede voorlichting van onze kant nodig en een zorgvuldige afweging van uw kant. Vandaar dat u deze schriftelijke informatie ontvangt. U kunt die rustig (her)lezen en in eigen kring bespreken. Ook daarna kunt u nog altijd vragen stellen aan de artsen die aan het eind van deze informatie genoemd staan.

*Achtergrondinformatie:*

Helaas houdt het gebruik van de catheter ook het risico in dat na verloop van tijd een infectie van deze catheter optreedt. Zo’n infectie kan ontstaan door het onbedoeld inbrengen van bacteriën in de catheter tijdens de toediening van vocht of geneesmiddelen. Recent onderzoek dat in het ErasmusMC werd verricht leerde dat, ondanks het toepassen van uitgebreide voorzorgsmaatregelen, 1 op 3 patiënten een catheterinfectie oploopt[[1]](#footnote-2). Niet alleen is koorts en toegenomen “ziek zijn” hiervan het gevolg maar een catheterinfectie maakt ook een langdurige behandeling met antibiotica noodzakelijk. Tenslotte is het in ongeveer de helft van de gevallen noodzakelijk de catheter te verwijderen om de infectie definitief te genezen .

In het Erasmus Medisch Centrum vindt momenteel onderzoek plaats naar het voorkomen van catheterinfectie (met name de ***Alcohol-Lock studie***). Dit onderzoek gebeurt met de goedkeuring van de Medisch Ethische Commissie van het ziekenhuis. De bedoeling van dit onderzoek zetten we hieronder even op een rij.

*Doel van het onderzoek:*

Met dit onderzoek willen we nagaan of het vullen van de catheter met 1 tot 2 milliliter alcohol gedurende 30 minuten per dag de kans op het ontstaan van een catheterinfectie vermindert. Deze toediening gebeurt door de verpleegkundigen van de afdeling. Ze vindt plaats zolang u de catheter behoudt en gebeurt dagelijks zolang u bent opgenomen in het ziekenhuis en 1 keer per week wanneer dit niet het geval is.

Alcohol is een goed en goedkoop ontsmettingsmiddel. Het is verder onschadelijk wanneer het, zoals in deze studie, in erg kleine hoeveelheden wordt toegediend. De hoeveelheid alcohol die dagelijks zal worden gebruikt is vergelijkbaar met één slok wijn of bier. Het uiteindelijke doel van dit onderzoek is de verschillende technieken om catheterinfectie tegen te gaan (waarvan het gebruik van alcohol er één is) te evalueren naar hun gemak, werkzaamheid en veiligheid. Op die manier hopen we in de toekomst zoveel mogelijk catheterinfecties te voorkomen.

*Wat houdt het onderzoek in voor u ?*

Wanneer u meedoet met de studie zal de apotheker van het ziekenhuis willekeurig beslissen of uw catheter op de gebruikelijke manier zal worden verzorgd en er dus geen alcohol zal worden gebruikt of dat u de extra behandeling met alcohol zal krijgen. Wanneer u geen alcohol krijgt toegediend wordt er in de plaats daarvan een fysiologische zoutoplossing gebruikt.

De arts die u behandelt noch de verpleegkundige wordt tijdens het onderzoek ingelicht over de behandeling die de catheter krijgt. Dit is de gebruikelijke manier om 2 behandelingen te vergelijken. Dit noemt men een dubbel blinde, placebo gecontroleerde studie. Aan het eind van het onderzoek wordt deze informatie pas vrijgegeven.

De kans dat u met alcohol behandeld zal worden is 1 op 2. Met andere woorden de helft van de patiënten die meedoen krijgt de nieuwe behandeling. Bij de andere patiënten worden de gebruikelijke procedures toegepast (met andere woorden wat er gebeurt indien u niet met de studie zou meedoen).

Er zijn voor u natuurlijk geen extra kosten aan de studie verbonden noch zijn er extra bloedafnames noodzakelijk.

*Vertrouwelijkheid (privacy)*

Uw persoonlijke gegevens zullen worden behandeld volgens alle wettelijke regelingen met betrekking tot vertrouwelijkheid. Door uw deelname aan de studie geeft u het recht aan de studieartsen om uw dossier in te kijken en de nodige gegevens te verzamelen. Uw naam en adres zullen in geen enkele van de wetenschappelijke verslagen die voortvloeien uit dit onderzoek vermeld worden. Tot uw persoon herleidbare onderzoeksgegevens kunnen slechts met uw toestemming door daartoe bevoegde personen worden ingezien. Deze personen zijn medewerkers van het onderzoeksteam, medewerkers van de Inspectie voor de Gezondheidszorg, en leden van de Medisch Ethische Toetsings Commissie Erasmus MC. Inzage kan nodig zijn om de betrouwbaarheid en kwaliteit van het onderzoek na te gaan. Onderzoeksgegevens zullen worden gehanteerd met inachtneming van de Wet Bescherming Persoonsgegevens en het privacyreglement van het Erasmus MC.

*Weigeren voor en tijdens het onderzoek*

Het is, zoals gezegd, niet precies bekend welke van de twee behandelingen van de catheter de beste is. Daarom heeft uw arts u verteld over het doel van dit onderzoek en u gevraagd om er aan mee te werken. U bent uiteraard vrij om uw medewerking aan dit onderzoek te weigeren. Als u besluit niet mee te doen, zal de catheter op de gebruikelijke “standaard” manier worden behandeld. Ook indien U nu toestemming geeft, kunt u die later zonder opgave van redenen weer intrekken. Wat u ook besluit, het zal geen consequenties hebben voor de verzorging en begeleiding van uzelf en uw familie. Indien u dit zou wensen heeft u ook het recht de verzamelde gegevens in te kijken.

*Mogelijke voordelen verbonden aan de studie:*

Er is een ***mogelijke*** ***voordeel aan uw deelname aan de studie*** verbonden. Met de nieuwe behandelingsmanier zal u misschien minder kans hebben om een catheterinfectie op te lopen. Alhoewel de onderzoekers dit natuurlijk hopen en verwachten werd dit tot nu toe echter nooit uitgezocht. Verder hopen we door uw deelname aan deze studie informatie te verzamelen die bij de behandeling van toekomstige patienten van pas zal komen.

*Mogelijke nadelen als gevolg van de studie:*

De ***kans op nadelen die een gevolg kunnen zijn van uw deelname aan de studie*** lijkt erg klein. Zoals gezegd is de hoeveelheid alcohol namelijk erg gering (één slok pils of wijn). Een allergische reactie op de toegediende alcohol is mogelijk indien u in het dagelijks leven om deze reden ook nooit alcohol gebruikt. Indien dit het geval is vragen we dan ook dit aan de onderzoekers te melden zodat we afzien van uw deelname. In een eerder onderzoek bij 28 kinderen over het gebruik van alcohol ter voorkoming van catheterinfectie werden geen noemenswaardige bijwerkingen vastgesteld.

*Schade*

De artsen betrokken bij deze studie zijn voor hun medische aansprakelijkheid verzekerd conform de wettelijke bepalingen inzake patientgebonden onderzoek.

*Nadere informatie*

Als u nog verdere vragen heeft dan kunt u die voorleggen aan uw behandelend arts of aan de bij het onderzoek betrokken artsen. Dit zijn:

dr. PJ Lugtenburg of dr. JK Doorduijn van de afdeling hematologie en dr. Bart JA Rijnders, dr. Siem de Marie en dr. M.C. Vos, bereikbaar via het secretariaat van de dienst microbiologie (tel. 010/4634405).

Als onafhankelijk arts kunt u raadplegen:

Dr. A. van der Geest, internist oncoloog. Tel. 010-4634897

Om er zeker van te zijn dat u deze patiënteninformatie heeft ontvangen en dat deze met u besproken is, verzoeken wij u bijgaand formulier gedateerd en getekend aan uw specialist te geven. Na ondertekening ontvangt u een kopie van het toestemmingsformulier.


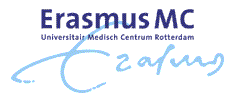


**Toestemmingsverklaring voor deelname aan het wetenschappelijk onderzoek:**

***Prevention of catheter-related infections with a daily alcohol Lock.***

***Randomised placebo controlled trial.***

Hierbij verklaar ik dat ik mondeling en schriftelijk ben geinformeerd over het gebruik van catheters in de bloedbaan en de behandeling van deze catheters. De verschillende behandelingen van deze catheters die in deze studie vergeleken zullen worden zijn met mij besproken.

Mij is gevraagd deel te nemen aan een onderzoek, waarbij de waarde van de behandeling met een alcohol slot ter voorkoming van infectie van de catheter zal worden bestudeerd. Ik heb begrepen dat door loting zal komen vast te staan of de catheter daadwerkelijk met alcohol zal worden behandeld. Ik heb voldoende gelegenheid gehad om vragen te stellen over de behandeling en het onderzoek. Deze vragen zijn bevredigend beantwoord.

Ik begrijp dat ik deelname aan dit onderzoek kan weigeren en dat ik mijn toestemming tot deelname te allen tijde kan intrekken. Ik stem vrijwillig toe met deelname aan het onderzoek.

Naam…………………………………………………………………………………………………………….

Adres…………………………………………………………………………………………………………….

Woonplaats………………………………………………….…………………………………………………..

Handtekening: ………………………………… Datum ……..…………….

Ondergetekende verklaart, dat de hierboven genoemde persoon zowel schriftelijk als mondeling over het bovengenoemde onderzoek geïnformeerd is. Hij/zij verklaart tevens, dat een voortijdige beëindiging van de deelname door bovengenoemde persoon, van geen enkel invloed zal zijn op de zorg die hem of haar toekomt.

Naam……………….…………………….. Functie ………..………………………….

Handtekening..……….……………………. Datum ……………………………………..

1. Nouwen JL et al (1999) Hickman catheter-related infections in neutropenic patients: insertion in the operating theater versus insertion in the radiology suite. J Clin Oncol 17:1304 [↑](#footnote-ref-2)
